# Supplementary material for: A historical evaluation of the disease avoidance theory of xenophobia
Source: PLoS One. 2023 Dec 27;18(12):e0294816. doi: 10.1371/journal.pone.0294816 (PMC10752500; doi:10.1371/journal.pone.0294816)
Supplement: S1 File — (DOCX) [file pone.0294816.s001.docx]

**Supplementary Materials**

**Appendix A**

**Distribution of the unfiltered target words and behaviors across the narratives of the explorers**.

Table 1. Term frequency of unfiltered physical avoidance words across the narratives.


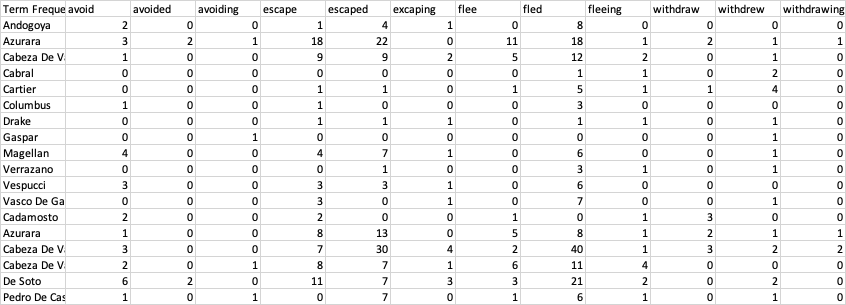


Table 2. Term frequency of unfiltered physical contact words across the narratives.


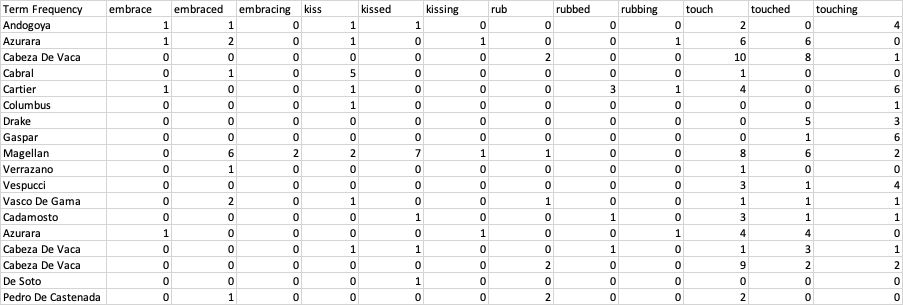


Table 3. Term frequency of unfiltered disgust-relevant words across the narratives


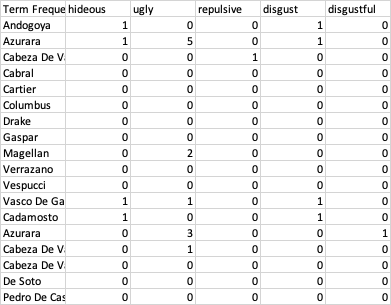


Table 4. Term frequency of unfiltered words that are antonyms or near-antonym of disgust across the narratives.


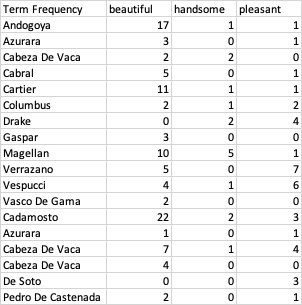


**Appendix B**

**The d algorithm.** The D score algorithm is calculated by the formulas below given by Caliskan et al (2017).

Let X and Y be two sets of target words of equal size, and A, B the two sets of attribute words.

Let cos ($\vec{a}, \vec{b})$ denote the cosine of the angle between the vectors $\vec{a} and \vec{b}$. The test statistics is given below.

$$\sum_{x\in X} s (x, A, B) -\sum_{y\in Y} s (y, A, B)$$

where $s (w, A, B) = {mean}_{a\in A\in} cos (\vec{w}, \vec{a}) - {mean}_{b\in B} cos (\vec{w}, \vec{b})$

Let {(X_i_, Y_i_,)}_i_ denote all the partitions of X $\cup$ Y into two sets of equal size. A two-tailed p-value of the permutation test is

Pr_i_ [s (X_i_, Y_i_, A, B) ≠ S (X, Y, A, B)]

The effect size is

$$\frac{{mean}_{x\in X} s(x, A, B) - {mean}_{y\in Y} s (y, A, B)}{{std-dev}_{w\in X\cup Y} s (w, A, B)}$$

**Appendix C**

**Keywords.** African, Central Africa, Central African, East African, West Africa, West African, South Africa, South African, Indians, Native Americans, Aborigines, Australia, Indigenous,

**Appendix D**

**Context words**. Since to a moderate degree the results would depend on the chosen context words, we followed a procedure described below to identify two sets of relatively opponent context words.

1. After a master context word such as “disgusting” within a category (disease avoidance words) was identified, we selected words that had at least 100 term frequency and were embedded with the master word to constitute our initial list of context words within that category. The initial list included the following words for the disease avoidance category: *Abject, Abominable, Detestable, Disagreeable, Disgusting, Grotesque, Hideous, Horrible, Horrid, Loathsome, Nasty, Repulsive, Revolting, Sickening, Sickly, Ugly, Unhealthy Unpleasant, Unwholesome*
2. We followed the same step above to generate a list of words that are relatively antonyms from the other set of context words. This list included the following words: *Agreeable, Attractive, Beautiful, Charming, Delightful, Delicious, Desirable, Enchanting, Exquisite, Finest, Gorgeous, Handsome, Healthy, Lovely, Pleasant, Pleasing, Pretty, Sweet, Wholesome*
3. Having created a matrix of the cosine similarities between all of the context words, we eliminated the words whose mean cosine similarities with the other words within the same set were negative or around zero.
4. We calculated a normalized difference (D) score for each word indexing how well it differs from the words in the opposite list by following steps:

Let Sets A and B have equal number of elements, n

Let $\vec{w}$_i_ be the vector of the i^th^ word in Set A, where i = 1, 2, …n

Let s_i_ be the mean cosine similarity score of word $\vec{w}$_i_ with all other words in Set A

Then,

$$s_{i}= \frac{\sum_{j=1}^{n} \cos(\vec{w}_{i} , \vec{w}_{j})}{n-1}$$

where j ≠ i

Let t_i_ be the mean cosine similarity score of the vector of word w_i_ with all word vectors in Set B

Then,

$$t_{i}= \frac{\sum_{j=1}^{n} \cos(\vec{w}_{i} , \vec{x}_{j})}{n-1}$$

where $\vec{x}$_j_ is the vector of the j^th^ word in Set B

Let ∆_i_ be the difference score of word w_i_

Then, ∆_i_ = s_i_ - t_i_

We normalized each difference score by the pooled standard deviation

Let 𝜎_A_ be the standard deviation of the mean cosine similarity scores in Set A

Let 𝜎_B_ be the standard deviation of the mean cosine similarity scores in Set B

Let P be the pooled standard deviation

Then, $P =\frac{\sigma_{A}+\sigma_{B}}{2}$

$$\Delta_{i}=\frac{s_{i}-t_{i}}{P}$$

We repeated the same process for each word in Set B to find the words embedded with each other strongly in Set B and those that differ from the words in Set A.

The final list of context words with the highest D scores in each set are below.

Words connoting activation of disease avoidance system: *abominable, detestable, disgusting, hideous, horrible, horrid, loathsome, nasty, offensive, repulsive, revolting, sickening, ugly*

Near antonyms of the disease avoidance words: *attractive, beautiful, charming, delicious, delightful, enchanting, exquisite, finest, handsome, lovely, pleasant, pretty, sweet*

**Group names**: An initial list of group names for the native groups also included African, Africans, Europeans, Inhabitant, Inhabitants, Native, Natives and Whites. The mean cosine similarity of these names did not differ from the names in the other set. We therefore removed these words from the list. We also excluded many ethnic group names (e.g., Sioux, Zanzibaris) since relatively fewer number of texts mentioned them. The number of narratives and the percentage of narratives group names were mentioned in the corpus are presented in table below.

| Group names | Number of Narratives | Percentage of Narratives |
| --- | --- | --- |
| Aborigines | 225 | 48% |
| Blacks | 207 | 44% |
| Bushmen | 110 | 24% |
| Hottentot | 108 | 23% |
| Hottentots | 100 | 21% |
| Indians | 311 | 67% |
| Kaffir | 100 | 21% |
| Kaffirs | 90 | 19% |
| Negro | 252 | 54% |
| Negroes | 304 | 65% |
| Tribe | 462 | 99% |
| Tribes | 402 | 86% |
| Zulus | 114 | 24% |
| American | 410 | 88% |
| Americans | 217 | 47% |
| British | 386 | 83% |
| Citizen | 154 | 33% |
| Citizens | 206 | 44% |
| Colonist | 113 | 24% |
| Colonists | 243 | 52% |
| English | 458 | 98% |
| Englishmen | 239 | 51% |
| European | 377 | 81% |
| Settler | 167 | 36% |
| Settlers | 348 | 75% |

**Morphology words**: *face, faces, hair, hands, lips, mouth, mouths, noses, nostrils, skin, skins, teeth, appearance, appearances, characteristics, complexion, complexions, countenance, countenances, expression, expressions, features, looking, looks, physiognomy, traits*

**Edibles names vs. Disease-causing object names**

We chose words with higher frequencies whether they are plural or singular of edibles and disease-causing objects to establish the validity of our context words.

**Fruit names**: *apple, apples, banana, cherry, figs, grapes, honey, mango, melons, oranges, peach, pineapples, strawberry*

**Disease-causing object names**: *dirt, dung, excrement, filth, flies, germs, insects, mosquitoes, parasites, rats, stench, ticks, vermin*

These two sets had similar frequencies, p = 0.5, (M _Fruits_ = 433, M _Disease agents_ = 572).

**Controlling Word Frequency.** Studies have shown that higher frequency words can lead to an overestimation of embedding bias (Ethayarajh et. al., 2019; van Loon et al., 2022). This presents a potential issue in word association tests since textual corpora often contain more frequent positive words than negative ones (Dodds et. al., 2015; Kloumann et al., 2012). Indeed, the context words exhibited significant differences in term frequency, p < 0.01 (M _Disgust_ = 442, M _Pleasant_ = 2763). While, differences in frequency between sets of context words can artificially generate embedding bias, the possibility of this occurrence also depends on the frequency of target words. When both target and context words have high frequencies, their likelihood of co-occurrence increases compared to situations where their word frequencies do not match (e.g., high vs. low). Conversely, when both target and context words have low frequencies, the algorithm assigns stronger weights to these rare occurrences, resulting in strong embeddings. In our case, since the group names in both sets had statistically similar word frequencies (p = 0.95, M _Natives_ = 4878, M _Nonnatives_ = 4756), we can expect their embeddings with the context words to be similar. To confirm this, we generated two sets of pseudo context words that varied drastically in word frequencies. One set consisted of the 13 most frequent words in the corpus (e.g., *the, of ...*) while the other set consisted of the 13 least frequent words. As expected, the cosine similarity of the groups names was higher with the high-frequency pseudo-context words than with the low-frequency pseudo-context words. However, both sets of group names exhibited statistically equivalent embedding bias with these pseudo context words due to their similar word frequencies (p = 0.70), Therefore, the difference in embedding bias observed between the native and nonnative groups with the context words is best explained by the semantics hidden in the corpus rather than mere frequency differences.
