# Supplementary material for: A historical evaluation of the disease avoidance theory of xenophobia
Source: PLoS One. 2023 Dec 27;18(12):e0294816. doi: 10.1371/journal.pone.0294816 (PMC10752500; doi:10.1371/journal.pone.0294816)
Supplement: Attachment — Submitted filename: Review of DiseaseAvoidenceandXenophobia.docx [file pone.0294816.s002.docx]

Review of “A Historical Evaluation of the Disease Avoidance Theory of Xenophobia”

The article is interesting as it examines the writings of European explorers to see if there is any support for the disease avoidance theory of xenophobia. It is well written. Although it is easy to understand the basis of the study, its discussion, and conclusions, I found the statistical and mathematical analysis difficult to understand—these aspects should be evaluated by reviewers familiar with these types of analyses.

I had a couple of small concerns when I read the paper. The document has no pagination, so I will reference my comments with quotes from the text as needed.

Results Section: The author conducted chi-squared tests based on data reported in Figure 1. It would be helpful to explain where the 45 and 36 degrees of Freedom come from and what difference is being tested. In Figure 1 there are only 5 words depicted and their frequencies. Thus, it is not easy to follow what is being tested.

Figure 3 is hardly readable where there is an overlap between words and the horizontal bars. Also, Figure 5 is not easy to read even when you enlarge it.

“occurrence of the word “repulsive”, but it was describing same-sex unions among Native…”

Comment: The word “but” in the above sentence needs clarification, or just remove the word, so as not to sound judgmental. It is not clear if the context of other words was considered in the analyses.

“Overall, our reading of the interactions between the indigenous people and European explorers between the 15th and 16th centuries did not reveal a pattern that would support the disease avoidance theory of xenophobia.”

Comment: It is not clear if this is based on “word counts” in the writings of European explorers or a content analysis of the writings or both.

It is impressive that the analyses did not support the disease theory of prejudice per these writings—but is it possible that the writings may have been biased on the part of explorers who minimized the use of such words so as not to appear biased? Is there a possibility of any political reasons for writing that the natives were welcoming of them, and hero-worshipped them as sent from heaven? On the other hand, the explorers were adventurers and may have had a more open attitude toward the natives and showed interest in them when they interacted with them.
