# Supplementary material for: A historical evaluation of the disease avoidance theory of xenophobia
Source: PLoS One. 2023 Dec 27;18(12):e0294816. doi: 10.1371/journal.pone.0294816 (PMC10752500; doi:10.1371/journal.pone.0294816)
Supplement: Attachment — Submitted filename: Letter to the editor for revision.docx [file pone.0294816.s003.docx]

Dear Dr. Rich,

I appreciate the time and effort, you and the reviewers put into the evaluation of my manuscript. I sincerely thank for the thoughtful comments and questions as they certainly enriched the quality of my work. I am addressing these comments and questions below:

Reviewer 1

The reviewer is absolutely correct in their comment regarding the long history of using historical data. As the reviewer indicates, calls for the use of historical data go back to the earlier years of psychology, especially in France (e.g., Meyerson). The use of historical data spanning several centuries with an explicit objective to test the historical validity of evolutionary theories is the novel idea in this study. This is especially true in the context of the first encounters between Europeans and newly discovered non-European populations and the disease avoidance theory of xenophobia. Nonetheless, I agree with the reviewer for their invitation to credit Simonton’s studies. Although the objective of using historical data was in a different line of research, Simonton is indeed a pioneer in using historical data in contemporary psychology. It is an oversight on my part to not cite his studies despite an earlier email communication with him. I revised the manuscript accordingly clarify that the manuscript here uses historical data to test the historical validity of the disease avoidance theory of xenophobia.

I added heading for Study 1 as the reviewer suggested.

Reviewer 2

1. I agree that presentation of the statistical analysis in study 1 was not clear. I have added a sentence to the method section and revised the results section to clarify which data were analyzed. Specifically, I rephrased the paragraph to explain that the degrees of freedom represent the total number of combined contact and contact avoidance behaviors, and the Chi Square analysis tested whether these words equally distributed between these two categories. The same approach was applied to combined disease avoidance words and their antonyms. Furthermore, I have adjusted the figure captions of figure 1 to clarify that asterisks next to each word indicate that conjugations of target words were also included in the analyses (e.g., disgust, disgusted, disgusting etc.)
2. To enhance clarity. I have removed the overlap between words and the horizontal bars in figure 3. In Figure 5, I conducted a modularity analysis enabling me to assign colors to nodes and edges, based on their respective communities. I increased the font size and presented the network and the section featuring words with the highest betweenness centrality index separately, making the figure more visible.
3. I revised the sentence in the discussion section, aiming to eliminate any judgmental tone. The intention behind the sentence was to emphasize that the term 'repulsive' did not pertain to the appearance of Native Americans.
4. I have rephrased the sentence of this paragraph to clarify that the word counts analysis of the texts did not support the disease avoidance theory of xenophobia. Furthermore, I have agreed to include a paragraph addressing the question raised by the reviewer. Indeed, there were several political and religious motivations that may have led European explorers to portray native populations positively. While these possibilities may apply to the verbal descriptions of the natives, they do not account for the frequent physical contacts observed between the groups, particularly given that these contacts were initiated by the natives themselves. Nonetheless, the question raised by the reviewer is significant for historical interpretation. The common personality trait shared among the explorers is intriguing and warrants a discussion on the generalizability of our findings. I have attempted to address these concerns in the general discussion.
5. Page numbers were provided
